# Does HIV adversely influence the outcome in advanced non-small-cell lung cancer in the era of HAART?

**DOI:** 10.1038/sj.bjc.6601111

**Published:** 2003-07-29

**Authors:** T Powles, C Thirwell, T Newsom-Davis, M Nelson, P Shah, S Cox, B Gazzard, M Bower

**Affiliations:** 1 Department of Oncology, Chelsea & Westminster Hospital, 369 Fulham Road, London SW10 9NH, UK; 2 Department of HIV Medicine, Chelsea & Westminster Hospital, 369 Fulham Road, London SW10 9NH, UK

**Keywords:** HIV, non-small-cell lung cancer, HAART

## Abstract

The objectives of the study are to assess the impact of HIV status on the outcome of patients with non-small-cell lung cancer (NSCLC) in the era of highly active antiretroviral therapy (HAART). Patients diagnosed with HIV-related NSCLC in the HAART era (since January 1996) were identified from a prospective single-centre lung cancer database. The clinicopathological characteristics and outcome of each HIV-positive patient were compared to three age- and stage-matched HIV-negative controls with NSCLC who were diagnosed over the same time period and treated in an identical manner. The results showed that the two groups had similar disease characteristics and received a similar amount of chemotherapy. The median overall survival of the two groups was the same (4 months, log rank *P*=0.55). None of the HIV-positive patients developed an AIDS defining illness or died of HIV during treatment or follow-up. In conclusion, in this cohort, HIV status does not influence the prognosis of advanced NSCLC. This suggests that the survival of patients with HIV-related NSCLC may have improved since the introduction of HAART, and this may be due to a decrease in HIV-related deaths.

HIV is associated with a small, but significant risk of developing lung cancer; particularly non-small-cell lung cancer (NSCLC) (Franceschi *et al*, 1998; Parker *et al*, 1998; Frisch *et al*, 2001). Before the introduction of highly active antiretroviral therapy (HAART), patients with HIV-related NSCLC had a worse outcome compared to age-matched HIV-negative controls (Sridhar *et al*, 1992; Vyzula and Remick, 1996; Tirelli *et al*, 2000). It has been suggested that this increased mortality was because the patients were younger, and had both more advanced and more aggressive disease compared to HIV-negative patients (Sridhar *et al*, 1992; Vyzula and Remick, 1996; Whooley *et al*, 1999; Skarin *et al*, 2001). Others have speculated that individuals with HIV-related lung cancer were offered suboptimal treatment due to concerns surrounding HIV-related comorbidity (Tirelli *et al*, 2000).

Since the introduction of HAART, the life expectancy of HIV patients has increased (Palella *et al*, 1998). This is largely due to a decrease in opportunistic infections; however, the outcome of a number of HIV-related malignancies has also improved (Tam *et al*, 2002). Currently, there are no controlled data regarding the outcome of patients with HIV-related lung cancer in the HAART era, despite reports that the incidence of HIV-related lung cancer may be rising (Bower *et al*, 2003).

In this study, we compare the treatment and outcome of HIV-positive and age-matched HIV-negative controls with advanced NSCLC in the HAART era.

## PATIENTS AND METHODS

The clinical details of patients with HIV-related NSCLC, diagnosed since the introduction of HAART in January 1996, were retrieved from a prospective single-centre lung cancer database. The HIV-negative control group was derived from the same database. For each HIV-positive case patient, three HIV-negative controls were identified who were of the same gender, had the same stage of lung cancer and were within 6 years of age of the case patient.

All patients included had histologically confirmed lung cancer, which for the cases was diagnosed after testing HIV seropositive. Routine lung cancer staging was undertaken in all patients using the American Joint Committee tumour-node-metastasis (TNM) system. The HIV-positive and control groups were treated in an identical manner using standard chemotherapy regimens (mitomycin, vincristine and cisplatin or gemcitabine and carboplatin, depending on the date of diagnosis). None of the patients had received prior chemotherapy or radiotherapy. The response to treatment was evaluated using the RECIST guidelines (Therasse *et al*, 2000) and toxicities were recorded according to the National Cancer Institute common toxicity criteria (CTC) version 2.0. Specifically age, date of cancer diagnosis, treatment, toxicity, response to treatment and survival were recorded. Survival was plotted according to the Kaplan–Meier method (Kaplan and Meier, 1958).

## RESULTS

Nine HIV-positive and 27 HIV-negative patients with NSCLC were identified between January 1996 and October 2002. The HIV-positive patients and HIV-negative controls had similar characteristics in terms of age, percent with stage IV disease and proportion with adenocarcinoma. However, HIV-positive patients appeared to have a worse performance status at presentation (Tables 1

**Table 1:** Comparison of clinicopathological properties of cases (HIV+) and controls (HIV−)

	**HIV positive**	**HIV negative**
*n*	9	27
Median age	45 years	48 years
	(range 32–58)	(range 30–55)
Adenocarcinoma	66%	70%
Stage IV disease	66%	70%
ECOG performance status <2 at presentation	22%	52%
	(range 1–4)	(range 1–4)
Treated with chemotherapy	88%	66%
Median number of cycles of chemotherapy	3.5	4
	(range 1–6)	(range 1–6)
Median overall survival	4 months	4 months
	(range 2–15+)	(range 1–24+)
Cancer-related deaths	77%	74%

and 2

**Table 2:** HIV-positive patients with NSCLC

	**Age (Years)**	**ECOG performance status**	**Histology**	**Stage**	**Site of metastasis**	**Treatment**	**Response**	**Survival (months)**
1	50	3	Squamous	IV	Bone	Chemotherapy	PD	2
2	58	2	Adeno	IV	Bone	Palliative		2
3	44	1	Squamous	IIIB		Chemotherapy	PR	5
4	48	4	Squamous	IV	Brain	Chemotherapy	PR	5
5	57	4	Adeno	IIIB		Chemotherapy	PR	3
6	45	1	Adeno	IIIB		Chemotherapy	PR	15+
7	43	3	Adeno	IV	Bone	Chemotherapy	PD	2
8	32	2	Adeno	IIIB		Chemotherapy	SD	8
9	43	2	Adeno	IV	Bone	Chemotherapy	SD	7

). The HIV patients were relatively asymptomatic with respect to their HIV disease, with a median CD4 cell count at presentation of 160 mm^−3^ (range 136–890 mm^−3^), and a median HIV viral load of 173 copies ml^−1^ (range <50–200 000 copies ml^−1^). Three patients had no detectable HIV viraemia and four had been diagnosed with an AIDS defining illness prior to the development of lung cancer.

The median overall survival in both groups was 4 months (range 2–15+ months for HIV positives *vs* 1–24+ months for HIV negatives). The 1-year survival for the HIV-positive patients was 11% compared to 22% for the HIV-negative group. The two groups received a similar number of cycles of chemotherapy (Table 1). Stable disease or a partial response was achieved in 50% of the patients treated with chemotherapy in both the groups of patients.

Chemotherapy was stopped early in three HIV-positive patients, two due to progression of disease and one due to chemotherapy toxicity. In all, 50% of HIV-positive patients treated with chemotherapy, developed grade 3 or 4 haematological toxicity; however, there were no chemotherapy-related deaths. Seven patients were on HAART at the start of treatment, two stopped due to concern surrounding interaction of chemotherapy and HAART. None of the HIV-positive patients developed an AIDS defining diagnosis or died of HIV-related disease during treatment or follow-up.

## DISCUSSION

Non-small-cell lung cancer occurs more frequently in the HIV population compared to HIV negatives (relative risk 4.5, 95% Confidence Interval: 4.2–4.8) (Frisch *et al*, 2001). Moreover, it has been reported that the incidence of NSCLC may be rising in people with HIV (Bower *et al*, 2003). The reason for the link between these two diseases is not clear; unlike most HIV-associated malignancies, no viral oncogene has been implicated in NSCLC. It has been speculated that the relationship is due to increased tobacco exposure in the HIV-positive population (Parker *et al*, 1998). In addition, pulmonary opportunistic infections and chronic immune suppression have both been implicated in the disease process; however, there is no clear consensus (Tirelli *et al*, 2000; Bower *et al*, 2003).

**Figure 1:**
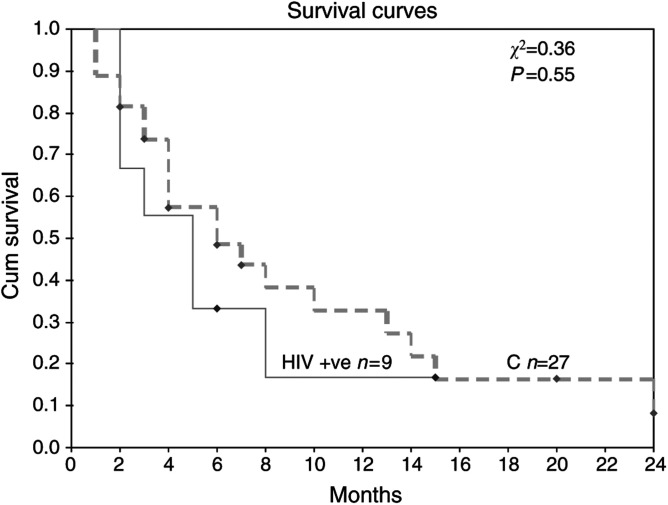
Kaplan–Meier overall survival duration curve from diagnosis of lung cancer according to HIV serostatus.

Before the introduction of HAART, HIV-related lung cancer patients had a worse outcome than HIV-negative age-matched controls (Sridhar *et al*, 1992; Vyzula and Remick, 1996; Tirelli *et al*, 2000) (Table 2). It was uncertain whether this was due to more aggressive lung cancer (Sridhar *et al*, 1992; Vyzula and Remick, 1996) or diagnostic delays and suboptimal treatment compared to HIV-negative controls (Tirelli *et al*, 2000). This controlled study compares the outcome of these patients since the introduction of HAART. It shows that the survival of the HIV-positive and HIV-negative groups is now the same, suggesting that the outcome of HIV-associated NSCLC has improved (Table 2, Figure 1

). Since in this study the groups were matched for NSCLC stage and the treatment protocols used were the same in both the groups, the results support the hypothesis that NSCLC is not more aggressive in the immunocompromised. The prior reports of worse survival in the HIV-positive patients may therefore have reflected excess deaths due to immunodeficiency rather than NSCLC. Indeed, there were fewer HIV-related deaths in this study compared to those in the pre-HAART era (0 *vs* 16–54%, respectively) (Hoffman *et al*, 2000; Tirelli *et al*, 2000) (Table 3

**Table 3:** Comparison with other studies in the pre-HAART era

	**Vyzula and Remick (1996)**		**Tirelli *et al* (2000)**		**Sridhar *et al* (1992)**		**Our study**	
**Reference**	**HIV +ve**	**HIV −ve control**	**HIV +ve**	**HIV −ve control**	**HIV +ve**	**HIV −ve control**	**HIV +ve**	**HIV −ve control**
Number	11	49	36	102	16	32	9	28
NSCLC (%)	100	100	86	81	94	97	100	100
Stage IIIB/IV (%)	75	NA	84	81	50	50	100	100
Median age (years)	44	<55	38	53	47	46	42	48
Median survival (months)	7.5	11	5	10	3	10	4	4
*P-*value for survival	0.003		0.0001		0.001		0.57	

NA=not applicable.

). Moreover, in this study, HIV-positive and -negative patients received equivalent amounts of chemotherapy. This does not appear to be the case for patients in the pre-HAART era, where dose modifications were common (Sridhar *et al*, 1992; Hoffman *et al*, 2000). These two factors may help explain the improved outcome of patients in this study.

Three of the four studies in the pre-HAART era were not well controlled. Two had a significantly older HIV-negative cohort (Hoffman *et al*, 2000; Tirelli *et al*, 2000), while the other was not matched for stage of disease (Vyzula and Remick, 1996). It may be that these confounding factors biased the results in favour of HIV negatives; however, there was a high degree of significance in all four studies (see Table 2). It has also been speculated that a smaller proportion of HIV patients with operable disease are offered surgery, resulting in worse outcome (Tirelli *et al*, 2000). This issue remains unresolved, as all of the patients in this study had advanced disease. The HIV-positive and -negative groups present in this study were remarkably similar, with a similar median age, proportion with adenocarcinoma and a similar amount of chemotherapy given. The HIV-positive patients did appear to have a poorer performance status than the negative group, which is associated with a worse outcome (Schiller *et al*, 2002).

Advanced HIV-related NSCLC is still associated with a poor outcome in the HAART era (median survival 4 months). However, this may reflect the advanced and aggressive nature of the disease in young people as a whole, rather than exclusively in people with HIV. It has been speculated that the high proportion of adenocarcinomas in the young, which is associated with early metastasis, may be responsible for this poor survival (Hoffman *et al*, 2000). There are no specific data on the median survival of patients with advanced NSCLC pre-HAART era; however, despite being incomplete, pooled data give a figure of approximately 2 months (*n*=23) (Sridhar *et al*, 1992; Vyzula and Remick, 1996; Alshafie *et al*, 1997).

It is of note that two of the patients in this study stopped HAART during or at the time of chemotherapy. The continuation of HAART is thought to reduce opportunistic infections and preserve immune parameters (Powles *et al*, 2002; Bower *et al*, 2003). However, if interactions between chemotherapy and HAART do occur, it may be worth considering a modification of the antiretroviral regimen rather than stopping the chemotherapy, as long-term prevention of HIV progression may be less of a priority in view of the poor prognosis of these patients. Prophylaxis against opportunistic infection is therefore crucial in this setting.

The data presented here suggest that the introduction of HAART may have improved outcome in advanced NSCLC in HIV-positive individuals. This may be due to decreased HIV-related mortality and an increased ability to tolerate treatment. These individuals may now have a similar outcome to HIV-negative people with the disease, provided that they are treated in a similar manner.
